# Monitoring of the Adult Patient on Venoarterial Extracorporeal Membrane Oxygenation

**DOI:** 10.1155/2014/393258

**Published:** 2014-04-03

**Authors:** Mabel Chung, Ariel L. Shiloh, Anthony Carlese

**Affiliations:** ^1^Division of Critical Care Medicine—The Jay B. Langner Critical Care Service, Department of Medicine, Montefiore Medical Center, Albert Einstein College of Medicine, Bronx, NY 10467, USA; ^2^Cardiothoracic Intensive Care Unit, Moses Campus, USA

## Abstract

Venoarterial extracorporeal membrane oxygenation (VA ECMO) provides mechanical support to the patient with cardiac or cardiopulmonary failure. This paper reviews the physiology of VA ECMO including the determinants of ECMO flow and gas exchange. The efficacy of this therapy may be determined by assessing patient hemodynamics and device flow, overall gas exchange support, markers of adequate oxygen delivery, and pulsatility of the arterial blood pressure waveform.

## 1. Introduction

Cardiopulmonary bypass (CPB) was used successfully for the first time in 1953 when Dr. John H. Gibbon repaired an atrial septal defect in an 18-year-old woman. Since then, technological improvements in extracorporeal life support have allowed for the development of a type of partial cardiopulmonary bypass called extracorporeal membrane oxygenation (ECMO). Not only has the technology of ECMO been brought out of the operating room into the bedside allowing clinicians to aid in the care of critically ill patients requiring pulmonary or cardiopulmonary support, but EMCO has also become remarkably portable and has allowed for intra- and interhospital transport of otherwise unstable patients. While ECMO support can be venovenous (VV) or venoarterial (VA), this paper aims to focus on the functional mechanics and the monitoring considerations in a patient with VA ECMO.

## 2. Indications/Benefits

**Figure 1:**
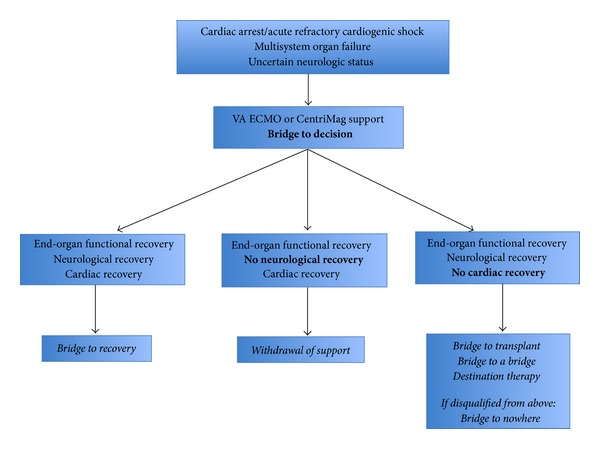
VA ECMO support after cardiac arrest provides hemodynamic stabilization, which allows time for therapeutic hypothermia and assessment of the neurologic status of the patient. If the patient achieves both neurologic and cardiac recovery, VA ECMO support will have functioned as a bridge to recovery and can subsequently be removed. If the patient does not recover neurologic function, VA ECMO support is typically withdrawn. If the patient awakens but does not have recoverable myocardial function, candidacy for heart transplant (bridge to transplant) and temporary (bridge to a bridge) or permanent (destination therapy) implantation of a ventricular assist device can be assessed. The neurologically intact patient that is disqualified from a heart transplant or ventricular assist device presents a dilemma that may be described as a bridge to nowhere. Figure adapted from [1].

VA ECMO provides both respiratory and hemodynamic support, in contrast to VV ECMO, which provides only respiratory support. VA ECMO is ideally placed in a patient with a reversible pathological process and is commonly placed in those with cardiogenic shock as well as those with other causes of hemodynamic instability refractory to medical management (Table 1). In the case of a myocardial infarction leading to cardiac arrest, peripheral VA ECMO can be placed quickly and can provide hemodynamic stabilization until the neurologic status of the patient is determined— a therapeutic strategy called bridge to decision [1, 2]. If the patient recovers neurologic function, VA ECMO support can be continued, allowing clinicians time to determine the suitability of the patient for myocardial recovery or as a candidate for transplantation or placement of a durable ventricular assist device. A decision tree for utilization of VA ECMO in the setting of cardiac arrest and uncertain neurologic status is outlined in Figure 1.

## 3. Limitations/Contraindications

VA ECMO provides partial hemodynamic support and can provide ventricular decompression, augmentation of perfusion pressure, and oxygenation and removal of carbon dioxide in the blood; however, it also increases the afterload against which the left ventricle (LV) works. The balance of the beneficial effect of decompression against the detrimental effect of increased afterload depends on the level of support and the state of the myocardium. Those on VA ECMO must be anticoagulated, the requirement of which must be weighed against the risk of bleeding. In addition, VA ECMO cannot be maintained on a long-term basis. Although there is no set time frame for device therapy, correction of physiologic derangements should occur within the first 24–48 hours. Recent studies have published durations of support ranging from 1.4 days to 11.5 days [2, 5–12]. VA ECMO is contraindicated in a number of conditions (Table 2).

## 4. Types of Cannulations/Circuit

**Figure 2:**
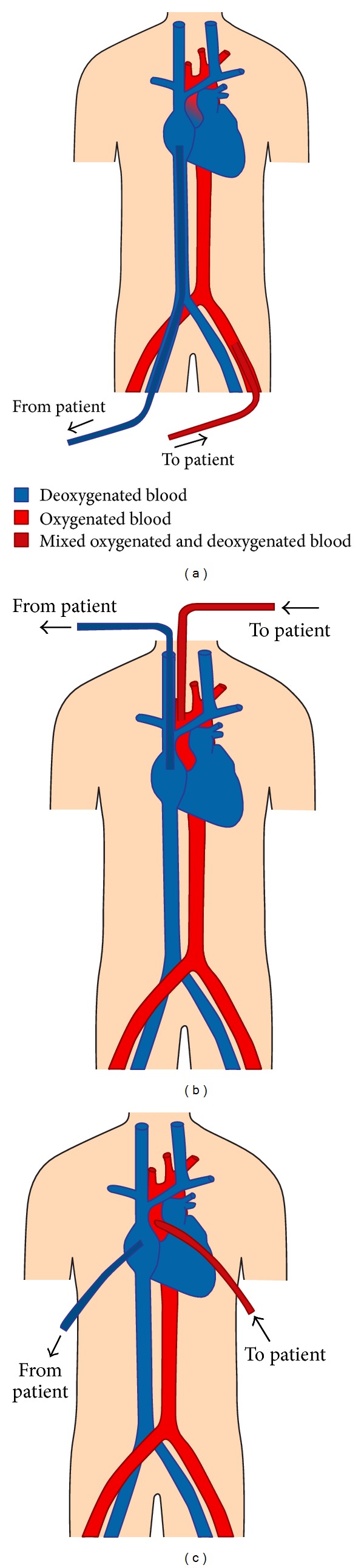
Illustrations of various VA ECMO cannulations. Diagram (a) depicts a femoral vein-femoral artery peripheral cannulation. Retrograde outflow from a femoral arterial cannula competes with anterograde cardiac output ejected from the left ventricle. In this situation, poor lung function results in the ejection of deoxygenated blood (blue) from the left ventricle, which mixes with oxygenated blood (red) from the ECMO circuit. The point of mixing (dark red) is located at the base of the aortic root, but will vary depending on the patient's heart function and ECMO flow. Poor lung function and good myocardial function in the context of a femoral-femoral ECMO cannulation may result in upper body hypoxemia (see text). Diagram (c) shows central cannulation with venous inflow drawn from the right atrium and arterial outflow pumped into the ascending aorta.

Cannulation for VA ECMO can be described as either peripheral or central (Figure 2) [3]. Peripheral cannulation can be accomplished either percutaneously or by cut-down, and typically utilizes the femoral or internal jugular vein for the venous (inflow) cannula and the femoral, axillary, or the carotid artery for the arterial (outflow) cannula. Peripheral cannulation, especially with the femoral vein and femoral artery, can be done quickly and on an emergency basis at the bedside. However, it often involves cannulas of smaller diameter than those used in central cannulation. Central cannulation, in contrast, requires a sternotomy or thoracotomy. It is frequently seen in the context of the inability to wean off CPB after cardiac surgery, as the cannulas used for bypass can be directly connected to the VA ECMO circuit. Central cannulation typically involves a venous cannula from the right atrium and an arterial cannula into the ascending aorta. The larger diameter cannulas allow for greater flow due to decreased resistance.

The fundamental components of a VA ECMO circuit include a venous inflow cannula, a pump, a membrane oxygenator/lung, and an arterial outflow cannula. The venous cannula withdraws blood at the level of the right atrium/vena cava. The blood is pumped through the membrane oxygenator allowing for oxygen uptake and carbon dioxide removal, and this arterialized blood is returned to the systemic circulation through an artery. Other components of the circuit may include a saturation sensor on the venous cannula to assess the mixed venous saturation (SvO_2_), a flow probe that clips onto to the arterial cannula to directly assess flow in liters/minute, a pre- and postoxygenator pressure monitor, a console whereby the speed of the pump can be adjusted, various access ports through which medications can be infused and blood samples withdrawn, a heat exchanger by which temperature can be controlled, and a bridge between the venous and arterial lines. Such a bridge allows blood to continue to circulate through the circuit after proximal clamping, which can be performed to test the effects of temporarily suspending ECMO support. The ability to circulate blood during clamping decreases the risk of stasis and thrombosis. An ECMO circuit may also include a venous reservoir or bladder located on the venous line prior to the pump to serve as an air bubble trap as well as a volume buffer. Centrifugal pumps (see following section) can generate substantial negative pressure at the venous inlet; thus, the presence of a reservoir can provide extra preload reserve to prevent cavitation of the cannulated vessel with resultant hemolysis. The bladder also allows for noninvasive monitoring of venous inlet pressure, although certain consoles may allow for pressure monitoring without the presence of a reservoir. Venous inlet pressures should not exceed negative 50 mm Hg.

## 5. Functional Mechanics: Flow and Gas Exchange

VA ECMO, as a form of partial cardiopulmonary bypass, provides 60–80% of the predicted resting cardiac output. The remaining 20–40% of venous return flows normally through the native pulmonary circulation. The cardiac output provided by the ECMO circuit (i.e., ECMO blood flow) is accomplished with one of two types of pumps—centrifugal or roller. This paper will focus on centrifugal pumps, which are more commonly used than roller pumps in the adult population. Centrifugal pumps like the CentriMag (Thoratec, Pleasanton, CA) propel blood forward with a magnetically levitated impeller that spins like a top.

The flow of blood through a VA ECMO circuit may be thought of as being governed by the modifiable variables of preload, afterload, and revolutions per minute (RPM) of the impeller as well as by the static variables of cannula length and diameter. Centrifugal pumps are preload dependent and afterload sensitive. The preload dependency of the centrifugal pump manifests as decreased flows with significant hypovolemia or with mechanical obstructive processes such as tamponade or tension pneumothorax. The centrifugal pump is also afterload sensitive. Decreased flows can occur with postpump obstructions such as thrombus in the oxygenator or kinks in the arterial cannula, as well as with excessive systemic vascular resistance (SVR) or mean arterial pressure (MAP). A decrease in the RPMs decreases flow through the circuit, while an increase in the RPMs, when not limited by preload, afterload, or circuit components, should cause an increase in the flow. Resistance to blood flow increases directly with cannula length and inversely with cannula diameter. Hence shorter and larger bore cannulas promote greater flow, while longer and smaller bore cannulas tend to limit flow. Circuit components are chosen to allow for at least 50–75 cc/kg/min of flow in adults (compared with 80 cc/kg/min for pediatric patients and 100 cc/kg/min for neonates). Larger patients may require additional inflow or outflow cannulas if adequate flows cannot be achieved with a given set of circuit components.

**Figure 3:**
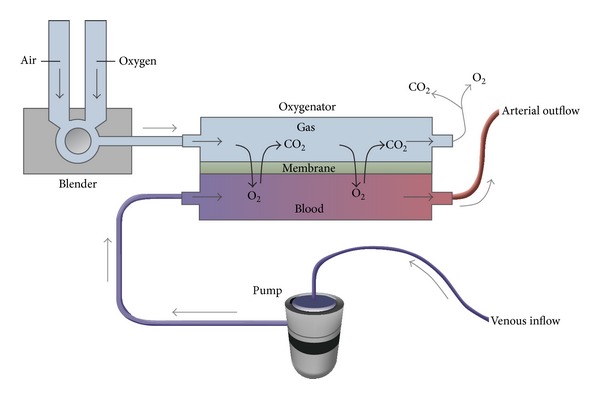
The oxygenator (also known as the membrane lung) is divided into a blood compartment and a gas compartment by a semipermeable membrane. The pump propels venous blood into the oxygenator and gas exchange occurs across the membrane as the blood interacts with fresh gas. After oxygenation and carbon dioxide removal, the arterialized blood is returned to the patient through an artery. A blender allows for adjustment of the fraction of delivered oxygen (F_D_O_2_). From [4]. Copyright © (2011) Massachusetts Medical Society. Reprinted with permission from Massachusetts Medical Society.

Gas exchange occurs in the membrane oxygenator (Figure 3) [4]. Extracorporeal venous blood is exposed to fresh gas (or sweep gas) that oxygenates and removes carbon dioxide. Both oxygen uptake and carbon dioxide removal depend on the presence of a diffusion gradient as well as on the available surface area of the semipermeable membrane. Oxygenation is affected by the fraction of delivered oxygen (F_D_O_2_) and the blood flow rate. A gas blender attached to the oxygenator mixes air and oxygen and allows for a range of F_D_O_2_. Increases in F_D_O_2_ will increase the partial pressure of oxygen in the blood (PaO_2_). In addition, increases in blood flow will also increase oxygenation as a greater volume of blood is exposed to the surface of the membrane. Augmentation of oxygenation only occurs up to a certain point after which the time for oxygen transfer becomes too short. Oxygenation is independent of sweep gas flow rate. In contrast to oxygenation, carbon dioxide elimination is dependent on sweep gas flow rate and is independent of blood flow (Table 3). A flowmeter regulates gas flow to the membrane. An increase in the sweep gas flow rate results in a decreased concentration of carbon dioxide in the fresh gas. This increases the diffusion gradient, promotes greater carbon dioxide elimination, and causes a decrease in the partial pressure of carbon dioxide in the blood (PaCO_2_). Carbon dioxide diffuses faster than oxygen because it is more soluble. As a result, it transfers approximately 10 times more efficiently than oxygen, sometimes necessitating the use of carbon dioxide enriched fresh gas in order to prevent hypocarbia. A comparison between pre- and postoxygenator blood samples should reveal an increase in PaO_2_ and a decrease in PaCO_2_. If such a change is not seen, membrane malfunction should be suspected.

Together, the blood flow and gas exchange of VA ECMO act as a surrogate heart and lung that supports end-organ function.

## 6. Monitoring

VA ECMO provides circulatory, oxygenation, and ventilatory support for the purpose of aiding with end-organ perfusion as well as to, potentially, provide myocardial rest. ECMO flows and the MAP should be monitored. The adequacy of gas exchange support must be verified by blood gases from an appropriately located arterial catheter. Markers of total body oxygenation—SvO_2_ and lactate—should be tracked to ensure adequate perfusion and oxygen delivery to the end-organs. And finally, the hemodynamic effects of VA ECMO upon the myocardium—beneficial or detrimental—may be gauged by following the pulsatility of the arterial waveform (Table 5).

### 6.1. ECMO Flows and MAP

VA ECMO flows should be monitored for changes. In the setting of a stable RPM, a drop in flow in a circuit with a centrifugal pump may be caused by decreased preload or excessive afterload. Decreases in preload may be secondary to hypovolemia or bleeding. The negative pressure generated by the pump in the hypovolemic state can cause hemolysis resulting in a rise in plasma free hemoglobin (significant if > 50 mg/dL) as well as a rise in lactate dehydrogenase (LDH). Spillage of free hemoglobin into the urine may result in a pink tinge to the urine. In addition, hypovolemia may also result in chattering— a low-frequency jerking or shaking movement of the cannulas due to a physical interaction between the inflow cannula and the vessel from decreased space. Hemolysis and chattering can occur independently of hypovolemia due to patient or cannula positioning or from excessive centrifugal pumps speeds (>3000 RPM) [13]. Inadequate preload may also be caused by mechanical obstructive processes such as tamponade, tension pneumothorax, and abdominal compartment syndrome. These processes decrease preload by restricting venous return and are typically associated with a rising central venous pressure (CVP). Drops in flow may also be caused by kinking of the venous cannula. Excessive afterload due to membrane oxygenator thrombus, a kink in the arterial cannula or a high SVR and MAP may also restrict flow through the VA ECMO circuit.

While maintenance of flows are crucial to the care of the patient on VA ECMO, attention must also be paid to the mean arterial pressure, as the end-organs require both a cardiac output as well as a perfusion pressure for optimal function. A goal MAP > 65 mm Hg may be used as a starting point but can be adjusted either lower or higher given individual circumstances. MAP should not exceed 90 mm Hg in order to limit afterload and to promote forward flow. Recall that

(1) $$\begin{array}{c} \text{MAP}=\text{CO}\times \text{SVR}, \end{array}$$

where CO is cardiac output and SVR is systemic vascular resistance. In the hypotensive patient, MAP may be increased by manipulating either CO or SVR. The total cardiac output of the body is composed of native cardiac output and VA ECMO flows. Thus, hypotension may potentially be corrected by increasing VA ECMO flows and its contribution to total CO. Assuming a centrifugal pump, this may be achieved by administering volume or by increasing the RPMs of the pump. If the problem is related to SVR, such as with septic shock, a vasoconstrictor may be needed to increase MAP, although this must be weighed against the effect of increased afterload and the increase in pressure work of the left ventricle (described in “Pulsatility” below).

### 6.2. Gas Exchange Support

As a partial cardiopulmonary bypass circuit, VA ECMO creates a separate circulation system parallel to the native circulation by siphoning a certain portion of venous return and reinfusing it as a contribution to the overall cardiac output of the body. The body's net oxygenation and ventilation depends on the interaction between the body's own capacity to oxygenate, ventilate, and perfuse (native lung function and cardiac output) and the contribution from VA ECMO in doing the same (membrane lung function and flow). The accurate interrogation of net gas exchange support depends on where the two systems converge. The location of the arterial ECMO cannula determines the point of convergence and the suitability of a particular arterial site for blood sampling or monitoring of SpO_2_.  Table 4 compares the different arterial cannulation sites with comments on the appropriate location of a peripheral arterial catheter for monitoring.

Cannulation of the right common carotid artery flushes well-oxygenated and ventilated blood down the right upper extremity. Thus, blood gases drawn from the right radial artery will not be reflective of net gas exchange support. Blood gases obtained distal to the location of mixing (e.g., the left radial artery) will be more accurate. Right radial arterial gases will not be accurate in right axillary artery cannulation, as well, due to its location relative to the ECMO cannulation site. Similarly, left radial arterial gases should be avoided in patients with left axillary artery cannulation. Direct aortic root cannulation allows for accurate blood gas analysis from any artery regardless of heart or lung function.

The preferred site of blood gas sampling with femoral artery cannulation is the right radial artery. When heart function is poor (with or without good lung function), retrograde ECMO flow should provide the vessels of the aortic arch with arterialized blood and allow good oxygen delivery to the coronary and cerebral circulations. Recovery of myocardial function pushes the mixing point of the two circulations more distally along the aorta causing native cardiac output to take over perfusion of the coronary and cerebral circulations. With good lung function, the coronary arteries, the innominate, and the right carotid artery will receive well-oxygenated blood. However, poor lung function in the setting of good myocardial function may cause these circulations to receive poorly oxygenated blood. In extreme cases, the patient's head may appear blue, while the lower extremities appear pink. Placement of a right radial arterial catheter, which interrogates oxygenation to the heart and brain, will allow for the detection of coronary and cerebral hypoxemia. This phenomenon is known by several descriptors including upper body hypoxemia, North-South Syndrome and Harlequin Syndrome.

Upper body hypoxemia may be addressed in several ways. The oxygen content of pulmonary venous blood may be augmented by adjusting ventilator settings, such as increasing the fraction of inspired oxygen (FIO_2_) and/or positive end-expiratory pressure (PEEP). Depending on the etiology, inadequate lung function may be addressed by performing recruitment maneuvers to decrease atelectasis, diuresing to decrease pulmonary edema, instituting antibiotic therapy for pneumonia, or utilizing thoracentesis or bronchoscopy for significant pleural effusions or secretions and mucous plugging. Upper body hypoxemia may also be remedied by manipulating aspects of VA ECMO support. VA ECMO flows can be increased in an attempt to better perfuse the aortic root with retrograde arterialized blood. In addition, the arterial outflow cannulation site can be switched from the femoral artery to the axillary or carotid artery. As they are in closer proximity to the aortic arch, these cannulation sites may be more effective in washing the root with oxygenated blood. However, cannulation of these smaller vessels will require a smaller cannula, which will decrease the maximum achievable flows. A VA-V ECMO circuit can also be created where a portion of arterialized blood from the arterial outflow cannula is diverted via the right internal jugular artery to the right heart. This enriches the blood traveling through the pulmonary circulation and to the left ventricle to provide better oxygen delivery to the coronary and cerebral circulations. Finally, if cardiac function has recovered sufficiently, VA ECMO can be converted to VV ECMO to provide only gas exchange support until the lungs fully recover function.

### 6.3. SvO_2_ and Lactate

SvO_2_, a measure of total body oxygenation and the balance between oxygen consumption and delivery, should be routinely assessed in the patient on VA ECMO. The body responds to a decrease in oxygen delivery (DO_2_) by increasing the extraction ratio (ER) of oxygen from the blood. Recall that

(2) $$\begin{array}{c} \text{ER}=\frac{{\text{VO}}_{2}}{{\text{DO}}_{2}}, \end{array}$$

where VO_2_ is oxygen consumption. Simplification of this equation results in the following:

(3) $$\begin{array}{c} \text{ER}=\frac{\left({\text{SaO}}_{2}-{\text{SvO}}_{2}\right)}{{\text{SaO}}_{2}}. \end{array}$$

Given a constant arterial oxygen saturation (SaO_2_), this equation reflects the inverse relationship of ER with SvO_2_. Assuming a SaO_2 _of 100%, a normal extraction of 25–35% results in a normal SvO_2_ of 65–75%. A state of high extraction will result in a low SvO_2_ of <65–75%; once oxygen extraction reaches a maximum of 50–60% and the SvO_2_ decreases to 40–50%, the body will begin to produce lactate due to the initiation of anaerobic metabolism.

A high ER or low SvO_2_ due to inadequate oxygen delivery may be secondary to inadequate ECMO support. Recall that

(4) $$\begin{array}{c} {\text{DO}}_{2}=\text{CO}\times {\text{CaO}}_{2}, \end{array}$$

where CaO_2_ is oxygen content. Given that the main components of CaO_2_ are hemoglobin (Hb) and SaO_2_ the equation may be simplified to

(5) $$\begin{array}{c} {\text{DO}}_{2}=\text{CO}\times \text{Hb}\times {\text{SaO}}_{2}. \end{array}$$

Thus, increasing VA ECMO flows (via increases in volume or RPM) to increase total CO will increase DO_2_ and may improve a suboptimal SvO_2_. Oxygen delivery can also be increased by red cell transfusion as well as by ensuring adequate arterial saturation. Because the anemic patient requires higher ECMO flows to achieve the same oxygen delivery, optimization of Hb prior to manipulation of flows may be desirable. A high ER or low SvO_2_ may also be caused by an increase in oxygen consumption; thus, interventions to decrease VO_2_ (antipyretics, cooling, antishivering agents, increasing sedation, etc.) can also be made concomitantly. A more complete description of oxygen delivery on VA ECMO may be delineated with this equation:

(6) DO2=(native  cardiac  output×CaO2  of  native  lung) +(ECMO  Flow×CaO2  of  membrane  lung).

A true SvO_2_ cannot be measured because venous return is split between the native and ECMO circulations. However, because the majority of blood flows through the VA ECMO circuit, it can be reasonably estimated by interrogating the venous cannula leading towards the membrane oxygenator either by blood gas analysis or a saturation probe to obtain a premembrane saturation. The minority of blood flows through the native pulmonary circulation. Hence, measurement of SvO_2_ from a pulmonary artery catheter may not be accurate.

Rearrangement of the equation for VO_2_ renders another equation for the variables that affect SvO_2_:

(7) $$\begin{array}{c} {\text{SvO}}_{2}={\text{SaO}}_{2}-\frac{{\text{VO}}_{2}}{\text{CO}\times \text{Hb}}. \end{array}$$

### 6.4. Pulsatility

VA ECMO provides rest to the myocardium by decreasing venous return and subsequently the volume work and wall tension of the heart. In addition, the decrease in preload decreases left ventricular end-diastolic volume (LVEDV) and pressure (LVEDP), thus promoting better coronary perfusion pressure due to a greater pressure gradient (coronary perfusion pressure = diastolic pressure – left ventricular end diastolic pressure). However, the return of blood into the arterial system increases afterload and the pressure work of the myocardium. The overall effect of the decrease in volume work and the increase in pressure work depends on the level of ECMO support as well as myocardial function and its response to these phenomena.

The ejection of blood flow out of the left ventricle generates a stroke volume and its arterial correlate, a pulse pressure. The absence of pulsatility in the arterial waveform in the setting of an appropriate level of support (60–80% of the predicted cardiac output allowing for the remaining 20–40% to pass through the lungs and heart) may be a sign of poor contractility and the heart's inability to overcome the increase in afterload despite the decrease in preload and volume work. Without pulsatility, blood within the left ventricle and at the aortic root may stagnate. In this situation, the risk of thrombus formation and subsequent embolic complications is increased. In addition, without the adequate ejection of blood, persistent venous return from thebesian and bronchial veins into the left atrium (LA) and ventricle will result in overdistension of the LV. This increase in LVEDV will cause an increase in LVEDP, which can compromise coronary perfusion pressure and cause additional ischemic damage to the myocardium. Finally, an increase in afterload in the setting of severe mitral regurgitation may result in left atrial hypertension and the transmission of pressures to the pulmonary system resulting in pulmonary edema and hemorrhage. Such a complication may occur even without preexisting valvular pathology, as the stasis of blood may cause left ventricular dilation and a functional mitral insufficiency due to dilation of the annulus. In this scenario, a pulmonary artery catheter may demonstrate an increase in the pulmonary capillary occlusion pressure. While assessment of the heart in a partially bypassed state can be challenging, transesophageal echocardiography may aid in confirming aortic valve opening as well as by providing an assessment of the left ventricular end-diastolic dimension.

VA ECMO flows can be reduced in an attempt to reduce afterload. However, this maneuver may not be possible if it compromises oxygen delivery and end-organ perfusion due to the inability of the heart to produce a compensatory increase in native cardiac output. Inotropic support can be instituted or escalated to increase contractility of the myocardium. In addition, afterload reduction with a vasodilator or intra-aortic balloon pump (IABP) may be implemented. An IABP brings the added benefit of improving coronary perfusion with balloon inflation during diastole. If these maneuvers fail to promote left ventricular ejection, decompression of the left ventricle may be necessary. Decompression may be accomplished by a percutaneous left atrial septostomy, which allows blood from the LA to drain down its pressure gradient into the right atrium (RA) to then be drained via the venous cannula. A catheter may also be placed into the LA through a transseptal puncture to facilitate drainage [14]. In addition, the left atrium or left ventricle can be directly cannulated allowing blood to be vented into the venous arm of the ECMO circuit. Finally, use of a left ventricular assist device such as the Impella 2.5 (Abiomed, Danvers, MA) to provide left ventricular decompression as well as forward flow has been described [15, 16].

Pulsatility is a dynamic property. Loss of pulsatility may signal worsening myocardial function, while the appearance of pulsatility or an improvement in pulse pressure may signal recovery. The differential diagnosis for loss of pulsatility also includes: 

*VA ECMO Flows That Are Too High*. The greater the ECMO flows, the more blood that drains into the circuit causing a greater decrease in LV preload, stroke volume, and pulse pressure. Total bypass, where the ECMO circuit takes over 100% of the cardiac output, creates a flat, nonpulsatile arterial tracing and signifies the lack of ejection of blood from the left ventricle.

*Hypovolemia/Mechanical Obstruction.* A decrease in intravascular volume or a mechanical cause of decreased venous return may result in a decrease in LV preload that leads to a decreased stroke volume and pulse pressure.

*RV Failure*. VA ECMO decreases RV preload and the volume work of the ventricle. However, pulmonary edema, lung collapse, or other parenchymal disease may cause hypoxic pulmonary vasoconstriction and may result in clinically significant pulmonary hypertension and an increase in RV pressure work. If this occurs to a significant extent, the right ventricle may be unable to deliver volume to the left side of the heart, resulting in a decrease in stroke volume and in pulsatility. In this scenario, pulmonary afterload reduction with nitric oxide or with inodilators such as milrinone and dobutamine (which will also provide inotropic assistance) may be beneficial. If systemic pressures allow, nitroglycerin or nitroprusside may also be utilized.

### 6.5. Rhythm

While it may be possible to maintain adequate hemodynamic and gas exchange support with VA ECMO during dysrhythmias that would otherwise be fatal, nonsinus rhythms such as ventricular fibrillation should be rectified due to the ineffective ejection of blood flow from the LV. Such arrhythmias should be addressed with direct current cardioversion, antiarrhythmics, pacing, or ablation.

### 6.6. Other Considerations

*Pre- and Postmembrane Oxygenator Pressures.* Exposure of blood to nonbiologic surfaces results in contact activation of the coagulation system resulting in a propensity to develop clot in the VA ECMO circuit. Premembrane pressures should be followed to assess for increases greater than 300–400 mm Hg. The postmembrane pressure can give useful context to the pre-membrane pressure. An elevated premembrane pressure in the setting of a normal post-membrane pressure suggests that the source of the increased resistance lies within the oxygenator. This scenario produces an increase in the difference between pre- and post-membrane pressures (i.e., an increase in Δ*P*, significant if >40 mm Hg) and may be indicative of thrombus on the membrane lung. If thrombus is suspected and is accompanied by a deterioration in gas exchange, the oxygenator may need to be replaced. An elevated pre-membrane pressure in the setting of an elevated post-membrane pressure suggests that the source of increased resistance is located downstream to the oxygenator, perhaps as a clot or kink in the cannula. 

*Partial Thromboplastin Time/Activated Clotting Time*. Heparinization is used to decrease the risk of developing clot. Adequate anticoagulation is monitored by partial thromboplastin time (PTT) and/or the activated clotting time (ACT). Heparin levels and antithrombin III levels may also be followed.

*Distal Ischemia*. Ipsilateral distal pulses as well as limb color and warmth should be assessed routinely with peripheral cannulations such as the axillary artery (arm ischemia) and the femoral artery (leg ischemia). In the case of femoral cannulations, a dorsalis pedis (DP) or posterior tibial (PT) distal perfusion cannula can be placed to promote perfusion to the lower extremity. Ischemic brain injury may occur as a consequence of carotid cannulation.

*Central Venous Pressure*. The CVP is altered by venous drainage during VA ECMO support; however, a rise in CVP in the setting of stable settings may be indicative of a mechanical obstructive process.

## 7. Recovery

Myocardial recovery on VA ECMO support is suggested by an increase in pulse pressure and by improved contractility on echocardiography. The ultimate test of myocardial recovery, however, is accomplished by assessing hemodynamic stability on minimal or no support. The RPMs can be decreased to achieve ~1 liter/min of flow or the VA ECMO cannulas can be briefly clamped. The native ventricle must be able to handle the full load of native cardiac output. If the myocardium has recovered, a decrease in or the temporary withdrawal of VA ECMO support should result in acceptable contractility on echocardiography and a stable MAP and CVP. Hypotension, a rising CVP, and a poorly contractile myocardium on echocardiography suggest inadequate recovery.

## 8. Conclusion

VA ECMO, a form of mechanical circulatory support, had its origins in the operating room as cardiopulmonary bypass and has evolved for use in the intensive care unit and beyond. Its purpose is simple—to replace some of the function of a failed cardiopulmonary system and to provide some rest to the myocardium. The successful achievement of such an aim, however, requires a thorough understanding of basic physiologic principles so that the weaknesses inherent to this therapy can be identified and rectified. As this technology continues to improve and becomes more accessible, knowledge of the principles underlying VA ECMO will only grow in importance to those involved in the care of the critically ill.

## Figures and Tables

**Table 1:** Indications for VA ECMO.

Refractory cardiogenic shock	
Myocardial infarction	
Myocarditis	
Primary graft failure following heart transplantation	
Postcardiotomy (failure to wean from CPB after cardiac surgery)	
Drug overdose resulting in profound myocardial depression	
Septic cardiomyopathy	
Peripartum cardiomyopathy	
Pulmonary embolism	
Recurrent dysrhythmias such as ventricular tachycardia/fibrillation	
Severe pulmonary hypertension	
Anaphylactic shock	
Trauma to major vessels or myocardium	
Massive hemoptysis or pulmonary hemorrhage	
Pre- or postprocedure circulatory support for high risk interventional procedures	

**Table 2:** Contraindications to VA ECMO.

Absolute contraindications	
Uncontrolled, active bleeding or other contraindication to anticoagulation	
End-stage, irreversible processes from which patient is not expected to recover (unless transplant candidate)	
(i) Cardiac disease	
(ii) Respiratory disease	
(iii) Neurologic disease	
Poor preexisting functional status or multisystem organ failure	
Unwitnessed cardiac arrest or prolonged cardiopulmonary resuscitation (>60 min)	
Aortic dissection	
Severe aortic valve regurgitation	
Other considerations	
Advanced age	
Renal or liver failure	
Active malignancy	
Morbid obesity	
Significant peripheral vascular disease	
Heparin-induced thrombocytopenia	

**Table 3:** Similarities and differences between VA ECMO oxygenation and ventilation.

Variable	Affects oxygenation	Affects CO_2_ elimination
Diffusion gradient	Yes	Yes
Membrane surface area	Yes	Yes
F_D_O_2_	Yes	No
Blood flow	Yes	No
Fresh gas flow rate	No	Yes

**Table 4:** Different sites of VA ECMO arterial cannulation and arterial catheter placement.

Arterial Cannula	Location of mixing	Arterial catheter site	Comments
Right common carotid artery	Aortic arch	Avoid right radial	Right radial blood gases inaccurate due to sampling of immediate downstream arterialized blood
Right axillary artery	Aortic arch	Avoid right radial	Right radial blood gases not reflective of blood to which rest of body is exposed
Left axillary artery	Aortic arch	Avoid left radial	Left radial blood gases not reflective of blood to which rest of body is exposed
Femoral artery	Between aortic root and descending aorta—exact location depends on native cardiac output and magnitude of retrograde flow	Preferred site right radial Avoid dorsalis pedis of cannulated limb	Right radial cannulation detects upper body hypoxemia (see text)
Aorta	Aortic root	Any	

**Table 5:** Summary of monitoring in VA ECMO.

	Monitor for	Treatment
Rhythm	Dysrhythmias such as ventricular fibrillation that may prevent ventricular ejection	Antiarrhythmics Cardioversion Pacing Ablation

MAP	Hypotension (MAP = CO × SVR) (i) Inadequate VA ECMO flow (ii) Inadequate SVR	(i) See “Flow” below (ii) Start vasoconstrictor

Pulsatility	Lack of pulsatility on arterial waveform caused by (i) poor myocardial function (ii) excessive VA ECMO support (iii) Inadequate preload (iv) RV failure May result in (i) thrombus (ii) myocardial ischemia (iii) pulmonary edema (assess CXR, wedge)	If poor myocardial function, consider: decreasing VA ECMO flow starting or increasing inotrope starting or increasing vasodilator IABP myocardial decompression

Flow (liters/min)	Low flows (assuming centrifugal pump) (i) Inadequate preload (a) Hypovolemia (may see hemolysis, chattering) (b) Mechanical obstructive (ii) Excessive afterload (thrombus, kink, SVR) (iii) Inadequate RPM	(i) Volume: crystalloid/colloid/transfusion Release of mechanical obstruction (ii) Exchange oxygenator, relieve cannula kink, vasodilator to decrease SVR (iii) Increase RPM

Gas exchange	Inadequate PaO_2_ inadequate or excessive CO_2_ elimination	
	(i) VA ECMO settings (a) F_D_O_2_ (b) VA ECMO flow (c) Sweep gas flow rate	(i) If hypoxemia, increase F_D_O_2_ or flow. If hypercarbia, increase sweep. If hypocarbia, decrease sweep or add CO_2_.
	(ii) Oxygenator function (a) Pre- and postmembrane pressures (b) Pre- and postoxygenator gases	(ii) Increased Δ*P* and inadequate arterialization of postoxygenator gases suggest oxygenator malfunction
	(iii) Upper body hypoxemia (femoral-femoral cannulation)	(iii) Increase pulmonary venous O_2_ content Adjust ventilator settings Treat etiology of pulmonary dysfunction Increase VA ECMO flow Change to axillary/carotid cannulation VA-V ECMO VV ECMO

Oxygen delivery: SvO_2_ and lactate	Decreased SvO_2_ and increasing lactate suggest inadequate oxygen delivery (DO_2_ = CO × CaO_2_) (i) VA ECMO flow (ii) Hemoglobin (iii) SaO_2_ Excessive oxygen consumption (ER = VO_2_/DO_2_) (i) Febrile (ii) Shivering	(i) Increase VA ECMO flow (ii) Transfuse (iii) Ensure adequate gas exchange (i) Antipyretics (ii) Consider agents such as meperidine or dexmedetomidine

Distal limb ischemia	Loss of pulses Cyanosis and coolness of limb	Femoral-femoral cannulation: DP or PT anterograde perfusion catheter

Anticoagulation	Adequate heparinization by PTT	

Temperature	Normothermia unless therapeutic hypothermia	
